# How to determine the course of preoperative chemotherapy for spinal tuberculosis: A single-center clinical study

**DOI:** 10.1097/MD.0000000000040232

**Published:** 2024-10-25

**Authors:** Qiang Liu, Long Ma, Zongqiang Yanga, Dawei Chu, Pengyu Yang, Kun Wang, Minyu Li, Jianping Zheng, Ningkui Niu, Jiandang Shi

**Affiliations:** a First Clinical Medical College of Ningxia Medical University, Yinchuan, Ningxia Hui Autonomous Region, China; b Department of Orthopedic, General Hospital of Ningxia Medical University, Yinchuan, Ningxia Hui Autonomous Region, China.

**Keywords:** clinical efficacy, complete lesion removal, preoperative chemotherapy, spinal tuberculosis

## Abstract

This study aims to explore the clinical efficacy and feasibility of preoperative 1-week chemotherapy for patients with spinal tuberculosis (STB) undergoing complete lesion removal. Clinical data of 76 patients with STB who underwent complete focal debridement in our hospital were collected from June 2020 to September 2023. The patients were divided into 38 cases of preoperative 1-week chemotherapy group (Group A) according to the length of preoperative chemotherapy, and 38 cases of preoperative 2 to 4-week chemotherapy group (Group B). Perioperative related, imaging, and laboratory examination indices as well as postoperative neurological function recovery, postoperative pain recovery, related complications, and clinical efficacy were analyzed to compare the clinical efficacy of the 2 groups. All patients successfully completed the treatment of stage I complete lesion removal combined with bone grafting fusion and internal fixation. The difference in erythrocyte setting rate and C-reactive protein at the same postoperative observation time between the 2 groups was not statistically significant (*P* > .05). The visual analogue scale scores of patients in the 2 groups decreased significantly with prolonged time, and the difference was statistically significant (*P* < .05). All patients achieved satisfactory clinical efficacy (*P* < .05). All patients achieved good clinical outcomes, the difference between the groups was not statistically significant (*P* > .05). The difference in incision healing rate at 3 months postoperatively and bone graft fusion rate at 6 months postoperatively was not statistically significant between the 2 groups (*P* > .05). The dissemination and recurrence of *Mycobacterium tuberculosis* were not statistically significantly different between the 2 groups after surgery (*P* > .05). In summary, with complete lesion clearance, 1 week of preoperative chemotherapy is feasible in patients with STB with varying degrees of neurological dysfunction.

## 1. Introduction

Spinal tuberculosis (STB) is a common extrapulmonary tuberculosis and accounts for 40% to 50% of bone and joint tuberculosis.^[1]^ The high tuberculosis burden in our country is more serious than before due to the outbreak of neo-coronary pneumonia.^[2,3]^ The Global Tuberculosis Report 2022 published by the World Health Organization on October 27, 2022 suggests that 10.6 million new cases of tuberculosis will occur globally in 2021, an increase of 4.5% from 2020; 1.6 million people will die of tuberculosis; and the number of patients with drug-resistant tuberculosis will increase by 3% in 2020.^[4]^

The concentration of antituberculosis drugs in the vast majority of STB foci fails to reach an effective bactericidal concentration due to the presence of sclerotic bone formed by STB infection^[5–7]^; long-term preoperative antituberculosis treatment not only fails to stabilize the condition but may also make *Mycobacterium tuberculosis* resistant to antituberculosis drugs. The destruction of spinal bone caused by tuberculosis infection causes the formation of complications, such as impaired mobility, osteoporosis, thrombosis, and pressure ulcers in patients with STB.^[8]^ As such, long-term preoperative chemotherapy causes patients to suffer from pain for a longer period of time before the operation. How to balance the duration of preoperative chemotherapy and postoperative efficacy has become an urgent problem for clinicians. In view of the many shortcomings of preoperative conventional chemotherapy, many scholars conducted studies to shorten the duration of preoperative STB chemotherapy. Scholars^[9,10]^ used 4 combinations of antituberculosis drugs for 1 to 2 weeks; when the symptoms improved, that is, erythrocyte setting rate (ESR) and C-reactive protein (CRP) was stable or had a tendency to decline, then surgery was performed; *M tuberculosis* dissemination was found. Alici^[11]^ treated 58 patients with STB with preoperative chemotherapy for 6 to 18 hours to save the neurological function prior to surgery. Xue Zhonglin^[12]^ operated on 16 patients with STB after 1 week of preoperative quadruple chemotherapy, and no recurrence was observed in the postoperative period. Ma^[13]^ performed tuberculosis debridement in 27 patients with STB after an antituberculosis regimen of 6 to 18 hours preoperatively, and there was no recurrence of STB in the postoperative period. Li Juan^[14]^ treated patients with STB with standardized antituberculosis drugs for 1 to 2 weeks preoperatively, and no cases of *M tuberculosis* dissemination were found. Yin^[15]^ et al treated 31 patients with STB with antituberculosis therapy for 3 to 17 days preoperatively, and no dissemination of *M tuberculosis* was found postoperatively. Fan^[16]^ designed a prospective clinical trial, and found that antituberculosis efficacy was comparable between the 2-week and the 4-week periods preoperatively.

In this work, we used a retrospective case–control study to collect the clinical data of 76 patients with STB who underwent complete lesion removal in our hospital. The patients were divided into groups with 1 and 2 to 4 weeks of preoperative chemotherapy. The clinical efficacy and feasibility of 1-week preoperative chemotherapy were evaluated by comparing various clinical observation indices between the 2 groups.

## 2. Information and methods

### 2.1. Inclusion and exclusion criteria

#### 2.1.1. Inclusion criteria

The inclusion criteria were as follows: (1) diagnosed with STB according to pathological examination and/or tuberculosis culture; (2) able to achieve complete focal clearance; and (3) not combined with active tuberculosis in other parts of the body.

#### 2.1.2. Exclusion criteria

The exclusion criteria included the following: (1) those who are unable to achieve complete focal clearance; (2) confirmed diagnosis of antituberculosis drug resistance during the patient’s treatment; (3) poor patient adherence; and (4) combination of severe liver and kidney dysfunction and immunity-related diseases.

### 2.2. General clinical information

Retrospective analysis of 76 patients with STB who were admitted to our department and underwent complete lesion removal from June 2020 to September 2023 were divided into the following according to the time of preoperative chemotherapy. Among 38 patients in the preoperative 1-week chemotherapy group (Group A), the following cases were recorded: 35 cases of low-grade fever, 30 cases of night sweating, 31 cases of fatigue, and 15 cases of poor nativity and lethargy; 38 cases of local pain in the lesion segments, and 25 cases of abscesses; 8 cases of thoracolumbar vertebral kyphosis; 8 cases of lesion, with 8 cases in thoracic vertebrae, 3 cases in thoracolumbar segment, 20 cases in lumbar vertebrae, 7 cases in lumbosacral segment; and no case involving single segment, 33 cases of double segment, and 5 cases of 3 segments and above. These patients were aged 49 ± 14.5 years (19–81 years) and had ESR of 437.28.74 ± 15.93 mm/h and CRP of 53.71 ± 22.88 mg/dL. Among 38 cases in the chemotherapy group (Group B) in the preoperative 2 to 4 weeks, the following cases were recorded: 32 cases of low-grade fever, 29 cases of night sweats, 27 cases of fatigue, and 27 cases of poor appetite and lethargy; 38 cases had localized pain in the diseased segments, and 26 cases had abscess; 11 cases had posterior convex deformity of the thoracolumbar spine; 11 cases had posterior convex deformity of the disease; 11 cases of thoracolumbar vertebrae with convex deformity; in terms of lesion sites, 10 cases in thoracic vertebrae, 3 cases in thoracolumbar vertebrae, 21 cases in lumbar vertebrae, and 4 cases in lumbosacral vertebrae; and 1 case involving a single segment, 29 cases of double segments, and 8 cases of 3 or more segments. These patients were aged 50 ± 17.5 years (15–75) and had ESR of 35.89 ± 11.88 mm/h and CRP of 52.08 ± 20.75 mg/dL. The preoperative general data of the 2 groups are shown in Table 1.

**Table 1:** Demographic and clinical characteristics of spinal tuberculosis patients enrolled in this study.

Items	Group A (n = 38)	Group B (n = 38)	Statistical values
Age (years)	49 ± 14.5 (19–81)	50 ± 17.5 (15–75)	*P* = .062
Gender (male/female)	23/15	21/17	*P* = .642
BMI	23.63 ± 1.65	23.76 ± 1.40	*P* = .415
Preoperative VAS scores	6.08 ± 1.23	5.74 ± 1.42	*P* = .340
Preoperative ESR (mm/h)	37.28 ± 15.93	35.89 ± 11.88	*P* = .150
Preoperative CRP (mg/L)	53.71 ± 22.88	52.08 ± 20.75	*P* = .434
Site of lesion (cases)			
Thoracic	8 (21.05%)	10 (26.32%)	*P* = .589
Lumbar	20 (52.63%)	21 (55.26%)	*P* = .818
Thoracolumbar	3 (7.89%)	3 (7.89%)	*P* = 1
Lumbosacral	7 (18.42%)	4 (10.53%)	*P* = .514
Involved segment			
1	0 (0%)	1 (2.63%)	*P* = 1.000
2	33 (86.84%)	29 (76.32%)	*P* = .237
≥3	5 (13.16%)	8 (21.05%)	*P* = .361
Surgical time (min)	229.7 ± 74.2	202.1 ± 64.7	*P* = .447
Intraoperative hemorrhage (mL)	422.3 ± 163.8	421.0 ± 171.0	*P* = .648

CRP = C-reactive protein, ESR = erythrocyte setting rate, VAS = visual analogue scale.

### 2.3. Grouping

All patients were treated with 300 mg of isoniazid, 300 mg of rifampicin, 750 mg of ethambutol, 750 mg of pyrazinamide, half an hour before breakfast in the morning in a single dose/day. According to the length of chemotherapy, the patients were divided into preoperative 1-week chemotherapy group (Group A) and preoperative 2 to 4-week chemotherapy group (Group B).

### 2.4. Surgical methods

All patients were subjected to general anesthesia with conventional tracheal intubation. The scope of lesion exposure and resection was decided according to the tuberculosis lesions shown in the preoperative imaging examination. According to the local pathological changes, complete lesion removal, bone grafting with support, internal fixation with instruments, spinal canal decompression surgery for patients with neurological dysfunction, and deformity correction surgery for patients with kyphosis were performed. The following cases were recorded: 16 cases of anterior thorough lesion removal and bone grafting, 38 cases of posterior internal fixation and anterior thorough lesion removal (canal decompression and deformity correction), and 22 cases of posterior (canal decompression and deformity correction) internal fixation and anterior thorough lesion removal and bone grafting. Lesion removal was performed from the anterior aspect of the vertebral body. After revealing the abscess, the pus was aspirated and scraped clean, and the site was washed repeatedly with saline. The diseased vertebrae and the proliferated granulation and scar tissue anteriorly or laterally in the upper and lower normal vertebrae as well as the normally covered muscles and segmental blood vessels were all separated. The segmental blood vessels were ligated, so the bone was completely exposed. After the complete removal of the lesion, the wound was repeatedly rinsed with saline. The defect left after resection was regular rectangular or square, and a large piece of iliac bone was cut and inlaid for implantation. Postoperative treatment with standardized antituberculosis drugs was continued.

### 2.5. Postoperative management

Changes in the vital signs of the 2 groups were recorded after the operation, and the sensation and motor status of both lower limbs was observed. The drainage tube was removed when the drainage flow was <20 to 50 mL in 48 to 72 hours after operation. The drainage tube for abscess cavity can be extended to 8 to 10 days. After 3 weeks of strict postoperative bed rest, the patients could wear a brace and move down to the ground. During the period of strict bed rest, the patients should pay attention to the functional exercise to prevent the occurrence of complications. All patients were treated with 2HRZS/2 to 7HRZS antituberculosis drugs for 2 to 7 months after the surgery, and the drugs were adjusted or stopped according to the review. Regular follow-up was done strictly according to the outpatient follow up card for patients with STB developed by our department.

### 2.6. Observation and evaluation indicators

The following indicators were monitored:

1. Perioperative evaluation indices: operation time, intraoperative bleeding, and final visual analogue scale (VAS) score of the 2 groups.
2. Laboratory indices: changes in ESR and CRP in preoperative, postoperative 1 month, 6 months, and 12 months.
3. Neurological function recovery: spinal cord neurological function recovery evaluated by Frankel grading during the follow-up before surgery and at the last follow-up.
4. Pain recovery: use of VAS to evaluate pain relief, with 0 being no pain and 10 being the worst pain.
5. Clinical efficacy: the efficacy evaluation criteria of Zhang Xifeng et al^[4]^ were used to evaluate the recovery of postoperative life and work ability; the evaluation criteria were divided into 4 grades: excellent, good, moderate, and poor; excellent: for medium and heavy manual laborers, able to engage in manual labor before surgery; for old and weak and light manual patients, able to recover to the self-care ability of daily life; good: although not able to recover to the working ability before the disease but able to completely take care of their own life; medium; loss of the working ability before surgery, only to maintain self-care, but tuberculosis has no recurrence; poor: tuberculosis lesion recurrence; observation of incision healing in the third postoperative month; and healing of the incision in the third month after the operation, and the standard of delayed healing of the incision. If the incision is red, swollen, and oozing after the operation, then it was not healed in 12 days, and the case is regarded as delayed healing. The dissemination of *M tuberculosis* was evaluated according to postoperative clinical manifestations, such as presence or absence of tuberculous meningitis, tuberculous pleurisy, and tuberculous peritonitis.
6. Cure and recurrence of STB foci. The criteria for cure were as follows: ① good condition, no local pain and pressure, no sinus tract formation; ② multiple reexamination results of ESR and CRP are normal; ③ imaging data showing the fully fused upper and lower interfaces of the implant bed; and ④ lack of new tuberculosis foci formed. The criteria for recurrence included the following^[17]^: the patients recovered well after the surgery, and all clinical indices returned to normal; however, new tuberculosis foci appeared in the original or other parts of the body after a period of time through imaging.
7. The imaging indicators were as follows: evaluation of bone graft healing based on 3D reconstruction of CT images; bone graft healing according to Moon criteria^[5]^: no loss of correction, no resorption of grafted bone, no resorption of grafted bone beds: appearance of obvious bone reshaping, appearance of continuous bone trabecular rearrangement between the grafted bone beds and grafted bone, and grafted bone coarsening.

### 2.7. Statistical methods

SPSS 26.0 software was used for data analysis. Measurement data satisfied normal distribution and were described by mean ± standard deviation ($\bar{x}\pm s$ ). Two-by-two comparisons were made by independent sample *t* test. Normal distribution was not satisfied. Median and quartiles were used for description. Count data were expressed as rate (%). Chi-square test was used for counting data (*X*^2^). Values at *α* = 0.05 and *P* < .05 indicated statistically significant differences.

## 3. Results

### 3.1. General information

Between June 2020 and September 2023, 76 patients underwent surgical treatment and received complete follow-up, with a follow-up time of 21.30 ± 3.17 months (17–26 months); at the last follow-up, patients in both groups had no symptoms of tuberculosis toxicity, such as low fever, night sweating, fatigue, no local pain, no abscess, no sinus tracts, and ability to return to their lives and work; the ESR and CRP had decreased to the normal levels, and the three-dimensional reconstruction of CT showed that the implanted bone was fused, and CT and MRI showed that the tuberculosis lesions were cured and fused. The three-dimensional reconstruction of CT showed that the bone grafts were fused, and the CT and MRI showed that the tuberculosis foci were cured and no new tuberculosis foci were formed. CT and MRI showed that the tuberculosis foci were cured, and no new tuberculosis foci were formed. The preoperative general data of the patients were comparable (*P* > .05, Table 1).

### 3.2. Perioperative evaluation indices

All 76 patients with STB successfully completed anterior complete removal of tuberculosis foci combined with anterior (posterior) bone graft fusion and internal fixation treatment. A total of 38 patients were placed in Group A and had an operation time of 229.7 ± 74.2 minutes (min), bleeding volume of 422.3 ± 163.8 mL. Meanwhile, 38 patients were placed in Group B and had an operation time of 202.1 ± 64.7 minutes (min), bleeding volume 421.0 ± 171.0 mL (mL). No statistically significant difference was found in the operation time and intraoperative bleeding between the 2 groups (*P* > .05, Table 1).

### 3.3. Laboratory indicators

**Figure 1.**
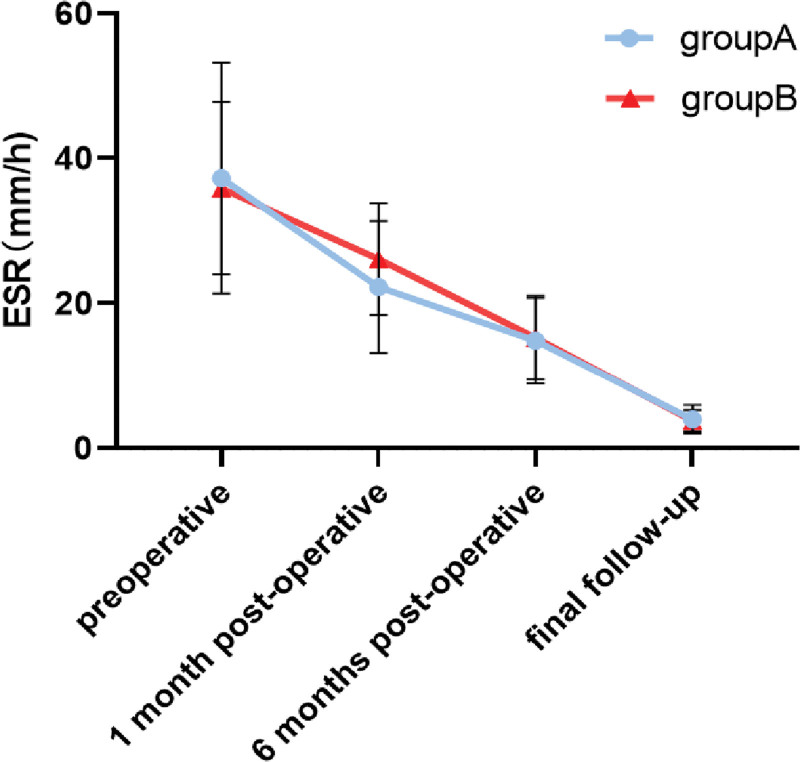
Trend of ESR over time in 2 groups. ESR = erythrocyte setting rate.

**Figure 2.**
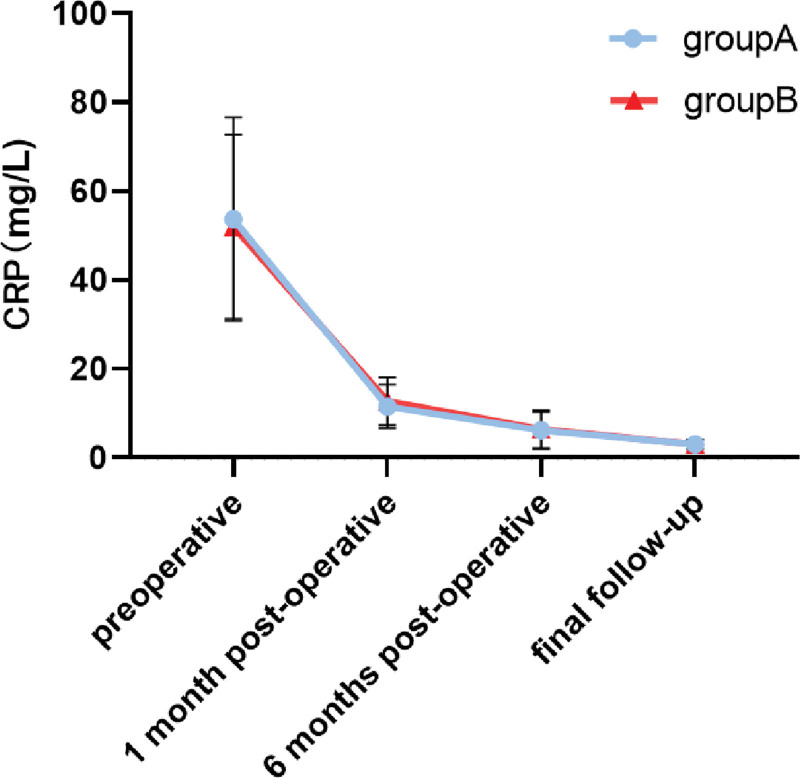
Trend of CRP over time in 2 groups of patients. CRP = C-reactive protein.

No statistically significant difference (*P* > .05) in ESR and CRP was found between the 2 groups in the preoperative period as well as 1 month, 6 months, and last follow-up. ESR and CRP were close to the normal levels in the 6 months after the operation and were normal in the last follow-up (Table 2, Figs. 1 and 2).

**Table 2:** Comparison of ESR and CRP between the 2 groups of patients at preoperative, 1 month and 6 months postoperatively and at the last follow-up ($\bar{x}\pm s$).

Items		Group A (n = 38)	Group B (n = 38)	*P*-values
Preoperative	ESR (mm/h)	37.28 ± 15.93	35.89 ± 11.88	.150
	CRP (mg/L)	53.71 ± 22.88	52.08 ± 20.75	.434
1 month postoperative	ESR (mm/h)	22.29 ± 9.09	26.13 ± 7.71	.247
	CRP (mg/L)	11.57 ± 4.90	12.76 ± 5.37	.567
6 months postoperative	ESR (mm/h)	14.87 ± 5.91	15.29 ± 5.78	.751
	CRP (mg/L)	6.21 ± 4.19	6.42 ± 4.31	.654
Final follow-up	ESR (mm/h)	3.99 ± 1.96	3.77 ± 1.52	.100
	CRP (mg/L)	3.05 ± 1.16	3.03 ± 0.97	.812

CRP = C-reactive protein, ESR = erythrocyte setting rate.

### 3.4. Postoperative pain recovery

**Figure 3.**
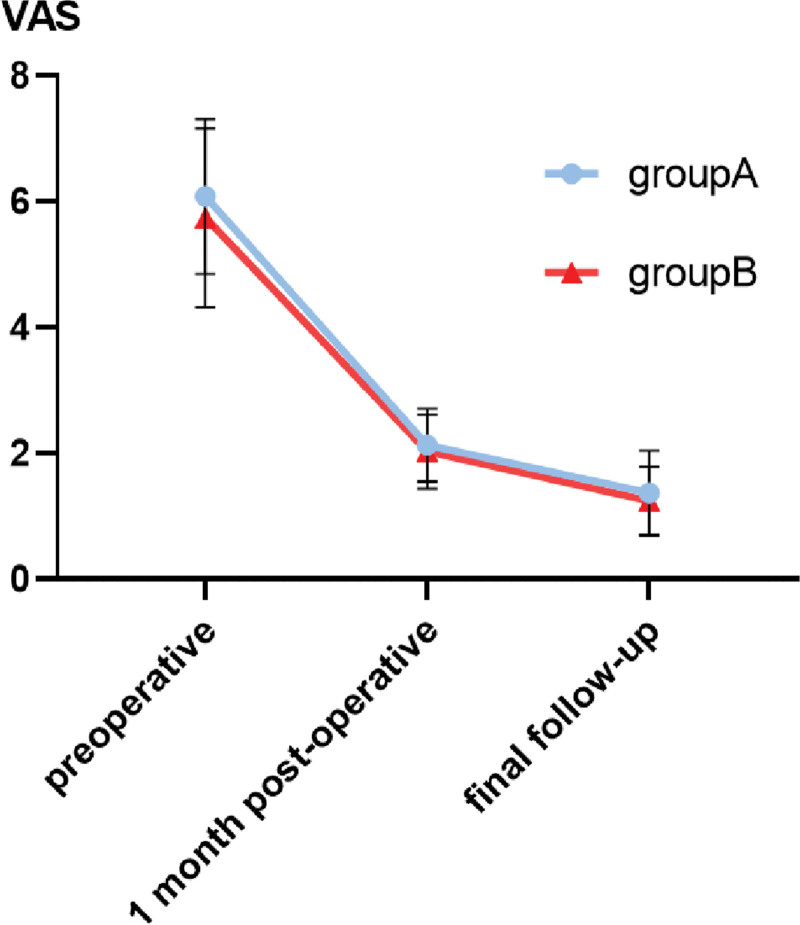
Trends in VAS scores over time for both groups of patients. VAS = visual analogue scale.

The VAS scores of patients in both groups decreased significantly with time, and the difference was statistically significant (*P* < .05), and the difference in VAS scores between the 2 groups was not statistically significant (*P* > .05, Table 3 and Fig. 3).

**Table 3:** Comparison of VAS scores between the 2 groups of patients.

VAS	Group A (n = 38)	Group B (n = 38)	*P*-values
Preoperative	6.08 ± 1.23	5.74 ± 1.42	.340
1 month postoperative	2.13 ± 0.58	2.03 ± 0.59	.059
Final follow-up	1.37 ± 0.67	1.24 ± 0.54	.088

VAS = visual analogue scale.

### 3.5. Postoperative neurological recovery

At the final follow-up, the AISA grades of neurological function of patients in the 2 groups were significantly higher than those before surgery, and the difference was statistically significant (*P* < .05). The difference in AISA grades between the 2 groups after the surgery was not statistically significant (*P* > .05, Table 4).

**Table 4:** Comparison of AISA grading of neurological function between the 2 groups of patients.

Groups	n	Preoperative ASIA grading	n	ASIA classification at last follow-up (cases)				
				A	B	C	D	E
Group A	38	A	3	0	0	1	0	2
		B	7	0	0	0	3	4
		C	25	0	0	1	3	21
		D	2	0	0	0	0	2
		E	1	0	0	0	0	1
Group B	38	A	2	0	0	1	0	1
		B	7	0	0	1	2	4
		C	27	0	0	2	0	25
		D	2	0	0	0	0	2
		E	0	0	0	0	0	0

### 3.6. Clinical efficacy

Both groups of patients achieved better clinical efficacy, with Group A and having excellent rate (86.79% and 84.28%, respectively). The difference in clinical efficacy between the 2 groups was not statistically significant (*X^2^* = 0.761, *P* > .05, Table 5).

**Table 5:** Comparison of clinical outcomes between the 2 groups.

Groups	Excellent (cases)	Good (cases)	Medium (cases)	Poor (cases)	Overall good rate
Group A (n = 38)	21 (55.26%)	11 (28.95%)	6 (15.79%)	0 (0%)	84.21%
Group B (n = 38)	19 (50.00%)	12 (31.59%)	7 (18.42%)	0 (0%)	81.58%

### 3.7. Imaging indicators

A total of 73 patients with STB obtained bony fusion in the implant area. Among 76 patients with STB at 6 months postoperatively, 37 cases belonged to Group A and 36 cases belonged to Group B, and the difference between the 2 groups was not statistically significant (*P* > .05, Table 6). Among 76 patients with STB, 2 cases in Group A and 3 cases in Group B were recorded; the difference in delayed healing of incision at 3 months postoperatively was not statistically significant (*P* > .05, Table 6). The postoperative bone healing times were 3.23 ± 0.77 (months) and 3.28 ± 0.81 (months) in the 2 groups, and the difference was not statistically significant (*P* > .05, Table 6). No deaths and spinal cord damages were recorded in 76 patients with STB after the operation, and none of them suffered from the dissemination of *M tuberculosis* and recurrence of tuberculosis in the postoperative period.

**Table 6:** Comparison of surgical outcomes between the 2 groups of patients.

Items	Group A (n = 38)	Group B (n = 38)
Incision healing at 3 months postoperatively	33/38 (86.84)	34/38 (89.47)
Bone graft fusion at 6 months postoperatively	37/38 (97.37)	36/38 (94.73)

## 4. Discussion

The complete removal of lesions and the reconstruction of spinal stability have been effectively guaranteed; however, the complete removal of lesions is not completely equal to the cure of lesions. Early, regular, whole course, appropriate dosage, and combined standardized application of antituberculosis drugs remain as the key to the cure of STB.^[18,19]^ Standardized antituberculosis drugs should be used for simple antituberculosis treatment or postoperative antituberculosis treatment^[20]^ and the role of different scholars’ suggested that short-course and ultra-short-course regimens for STB play a role in cure.^[21,22]^ Selecting preoperative chemotherapy regimen has no guidelines, and no basic medical research can be used to confirm the length of preoperative chemotherapy for STB; as such, so the information given in almost all studies on the timing of surgery for STB is ambiguous. Although 2 to 4 weeks of preoperative chemotherapy is mainly advocated in clinical practice, this has no theoretical basis.^[16]^

For patients with indications for surgery, the purpose of preoperative application of antituberculosis drugs is to basically control *M tuberculosis* and tuberculosis infection, stabilize the patient’s condition, reduce the patient’s symptoms associated with tuberculosis toxicity in a short period of time. This technique is also used to prepare the conditions for the smooth implementation of subsequent tuberculosis lesion removal surgery and to avoid systemic dispersal of *M tuberculosis* and recurrence caused by the surgery; however, completely killing local *M tuberculosis* is not required.^[23]^ Hongqi Zhang^[24]^ detected the DNA content of *M tuberculosis* in peripheral blood by polymerase chain reaction technology and reported that patients treated with antituberculosis medication for <2 weeks before the operation and whose ESRs were higher than the normal value were treated with tuberculosis foci removal surgery. The experiments did not show any dissemination of *M tuberculosis* in vivo. In addition, shortening the preoperative chemotherapy time of patients cannot only reduce the perioperative time of patients and early surgical intervention but also reduce the pain of patients, reduce their mental pressure, and avoid or reduce the pulmonary infection, decubitus ulcers, osteoporosis, muscular atrophy, thrombosis and other complications caused by STB patients’ prolonged bed rest; as such, this process is crucial for the rapid recovery of patients.^[25]^ Scholars^[26]^ believe that surgery should be performed as early as possible to ensure surgical safety as well as restore patients’ neurological dysfunction and improve their quality of life.

STB has many factors that affect efficacy, other than chemotherapy and surgery.^[27]^ For example, whether it is combined with tuberculosis in other parts of the body, whether it is combined with liver and kidney dysfunction, and whether tuberculosis drug resistance exists. Therefore, on the basis of standardized chemotherapy and thorough lesion removal surgery, strict control of other unfavorable factors can ensure the early cure of tuberculosis. Therefore, the implementation of preoperative 1-week chemotherapy program needs to strictly grasp the indications. This study concludes that 1-week preoperative chemotherapy is suitable for: (1) complete lesion removal; (2) simple STB, which cannot be combined with active tuberculosis in other parts of the body; (3) severe pain or obvious neurological symptoms, which cannot tolerate prolonged preoperative antituberculosis treatment; and (4) paraplegia or incomplete paraplegia can generally decide the time of preoperative antituberculosis treatment depending on the degree and location of paraplegia, such as cervical and thoracic tuberculosis with paraplegia; emergency surgery is feasible when necessary.

In this study, we analyzed and compared the clinical efficacy and related indices of patients with STB who underwent complete lesion removal and bone grafting after 1 week of preoperative chemotherapy and 2 to 4 weeks of preoperative chemotherapy. The VAS scores of patients in both groups decreased significantly with the extension of time (*P* < .05), but the differences in VAS scores at different time points between the 2 groups were not statistically significant (*P* > .05). At the final follow-up, the symptoms of tuberculosis toxicity and local pain disappeared in 55 patients; both groups achieved better clinical efficacy, and the difference between them was not statistically significant (*P* > .05). At the final follow-up, the neurological function of the 2 groups showed significant improvement. At the 6-month postoperative review, most of the bone grafted areas of patients in the 2 groups obtained osseous fusion, and the difference between the 2 groups was not statistically significant (*P* > .05). Recurrence of tuberculosis, delayed healing of postoperative incision, and dissemination of *M tuberculosis* were not observed in patients of the 2 groups after the operation. The above observational indices confirmed that the postoperative efficacy was comparable between the 2 groups, but the preoperative suffering time of Group A was shorter than that of Group B. Hence, under the premise of strictly grasping the indications, the 1-week preoperative antituberculosis chemotherapy program can be used for the treatment of STB, and excessively pursue the duration of preoperative antituberculosis treatment for 2 to 4 weeks or even longer is not necessary.

In conclusion, the 1-week preoperative chemotherapy regimen is feasible for the treatment of STB and can cure STB with satisfactory efficacy. Compared with 2 to 4 weeks of preoperative chemotherapy, the 1-week regimen has the advantages of higher efficiency, lower treatment costs and medical resources, and shortened duration of preoperative pain.

Although the results are satisfactory, several shortcomings should be noted. This study adopted a retrospective case–control design, and the evidence level of the case study is not high; the study was also conducted in a single center, wherein fewer cases with long-term follow-up are available.

## Author contributions

**Conceptualization:** Qiang Liu, Zongqiang Yang, Jiandang Shi, Ningkui Niu.

**Data curation:** Qiang Liu, Long Ma, Zongqiang Yang, Dawei Chu, Pengyu Yang, Kun Wang, Minyu Li, Jiandang Shi, Jianping Zheng, Ningkui Niu.

**Formal analysis:** Qiang Liu, Zongqiang Yang, Dawei Chu, Pengyu Yang, Kun Wang, Minyu Li, Jiandang Shi, Jianping Zheng, Ningkui Niu.

**Funding acquisition:** Zongqiang Yang, Dawei Chu, Kun Wang, Jiandang Shi.

**Investigation:** Long Ma, Zongqiang Yang, Jiandang Shi.

**Resources:** Long Ma, Pengyu Yang, Kun Wang, Jianping Zheng, Ningkui Niu.

**Software:** Qiang Liu, Long Ma, Pengyu Yang, Kun Wang, Jianping Zheng.

**Supervision:** Jiandang Shi, Ningkui Niu.

**Validation:** Long Ma, Minyu Li, Jiandang Shi, Jianping Zheng.

**Visualization:** Qiang Liu, Jianping Zheng.

**Writing – original draft:** Qiang Liu, Minyu Li.

**Writing – review & editing:** Qiang Liu.
